# Identification of the conjugative and mobilizable plasmid fragments in the plasmidome using sequence signatures

**DOI:** 10.1099/mgen.0.000459

**Published:** 2020-10-19

**Authors:** Zhencheng Fang, Hongwei Zhou

**Affiliations:** ^1^​ Microbiome Medicine Center, Department of Laboratory Medicine, Zhujiang Hospital, Southern Medical University, Guangzhou, 510280, PR China; ^2^​ Center for Quantitative Biology, Peking University, No. 5 Yiheyuan Road Haidian District, Beijing 100871, PR China; ^3^​ State Key Laboratory of Organ Failure Research, Southern Medical University, Guangzhou, PR China

**Keywords:** plasmidome, plasmid transmissibility, deep learning, sequence signatures, information theory

## Abstract

Plasmids are the key element in horizontal gene transfer in the microbial community. Recently, a large number of experimental and computational methods have been developed to obtain the plasmidomes of microbial communities. Distinguishing transmissible plasmid sequences, which are derived from conjugative or at least mobilizable plasmids, from non-transmissible plasmid sequences in the plasmidome is essential for understanding the diversity of plasmids and how they regulate the microbial community. Unfortunately, due to the highly fragmented characteristics of DNA sequences in the plasmidome, effective identification methods are lacking. In this work, we used information entropy from information theory to assess the randomness of synonymous codon usage over 4424 plasmid genomes. The results showed that for all amino acids, the choice of a synonymous codon in conjugative and mobilizable plasmids is more random than that in non-transmissible plasmids, indicating that transmissible plasmids have different sequence signatures from non-transmissible plasmids. Inspired by this phenomenon, we further developed a novel algorithm named PlasTrans. PlasTrans takes the triplet code sequences and base sequences of plasmid DNA fragments as input and uses the convolutional neural network of the deep learning technique to further extract the more complex signatures of the plasmid sequences and identify the conjugative and mobilizable DNA fragments. Tests showed that PlasTrans could achieve an AUC of as high as 84–91%, even though the fragments only contained hundreds of base pairs. To the best of our knowledge, this is the first quantitative analysis of the difference in sequence signatures between transmissible and non-transmissible plasmids, and we developed the first tool to perform transferability annotation for DNA fragments in the plasmidome. We expect that PlasTrans will be a useful tool for researchers who analyse the properties of novel plasmids in the microbial community and horizontal gene transfer, especially the spread of resistance genes and virulence factors associated with plasmids. PlasTrans is freely available via https://github.com/zhenchengfang/PlasTrans

## Data Summary

PlasTrans is freely available via https://github.com/zhenchengfang/PlasTrans. The accession numbers of the genomes associated with this study are listed in Table S1 (available in the online version of this article).

**Impact Statement**

In recent years, a large number of studies on the plasmidome of microbiome communities have emerged, but bioinformatic tools for plasmidome analysis are severely lacking. This work addresses a challenge associated with plasmid characterization, more precisely, the identification of transmissible plasmid sequences in plasmidome data. We showed that because of genome amelioration, transmissible plasmids contain different sequence signatures from non-transmissible plasmids, and we further developed PlasTrans to distinguish transmissible plasmids using sequence signatures based on deep learning. As the first tool for transmissible plasmid fragment identification, PlasTrans may be a powerful tool that will improve biologists' understanding of the properties and diversity of the plasmidome, and the mechanism through which plasmids regulate microbial communities.

## Introduction

Because of the important role of plasmids in the regulation of microbial communities, a large number of experimental and computational methods that obtain the plasmidome of microbial communities have been developed to help biologists better understand the functions of plasmids. The commonly used experimental methods include exonuclease treatment, phi29 DNA polymerase amplification [1], transposon-aided capture (TRACA) [2] and exogenous plasmid isolation [3, 4]. Additionally, many computational tools for sequence classification and assembly that identify plasmid sequences from chromosome-derived sequences in metagenomic data have been developed, such as cBar [5], PlasFlow [6], PPR-Meta [7], PlasClass [8], Recycler [9] and metaplasmidSPAdes [10]. These approaches have facilitated the discovery of a large number of novel plasmid sequences. However, we lack a series of effective plasmidome-specific bioinformatic tools, such as tools for plasmid classification and host prediction, for downstream analysis to better understand plasmidome function.

It has been estimated that approximately half of the isolated plasmid genomes are transmissible, while the rest are non-transmissible [11]. To better understand the diversity of plasmids, plasmid–host interactions and plasmid-assisted horizontal gene transfer in microbial communities, it is important to distinguish the transmissible plasmids (i.e. conjugative plasmids and non-conjugative but mobilizable plasmids) and non-transmissible plasmids in the plasmidome. It is generally thought that transmissible plasmids contain the relaxase gene and origin of transfer (*oriT*), while non-transmissible plasmids do not contain these elements [11, 12]. Therefore, with complete plasmid genomes, the classification of these two types of plasmids can be easily performed by searching for the relaxase gene or *oriT*. Recently, a pipeline named the MOB-suite, which can identify transmissible plasmids primarily by searching for the relaxase gene and *oriT*, has been developed [13].

Unfortunately, such marker gene-based methods work well for complete genomes but do not work for plasmidomes derived from microbial communities. Because of specific sequence features, such as the existence of a large number of repeated regions and shared genes, the sequence assembly of plasmid-derived reads in metagenomic data is much more difficult than that for chromosome-derived reads [9]. For example, a study on the bovine rumen plasmidome showed that only 36.8 % of reads could be assembled into 5771 contigs with a mean length of 469 bp [1]. This indicates that most highly fragmented DNA do not contain the marker genes for identification. Additionally, the plasmidome may contain a large number of novel plasmids. Our previous work estimated that more than half of the reads from the plasmidome could not be mapped to the RefSeq plasmid database [14]. Therefore, it is essential to develop a novel method that bypasses the search for related marker genes and sequence alignment against known databases to identify transmissible plasmid sequences in the plasmidome.

In this work, we first proposed the hypothesis that transmissible and non-transmissible plasmids contain different sequence signatures, and this hypothesis was then verified for 4424 plasmid genomes using information theory. This shows that it is possible to identify transmissible plasmid DNA fragments by using sequence signature information instead of searching related marker genes. Inspired by this phenomenon, we developed PlasTrans, which uses a convolutional neural network deep learning technique to efficiently extract the more complex and abstractive sequence signatures from plasmid DNA fragments and identify the DNA derived from transmissible plasmids. Tests show that PlasTrans can achieve high accuracy for plasmid DNA fragments of different lengths.

## Methods

### Randomness of synonymous codon usage

Because of genome amelioration, plasmids adjust their sequence signatures based on their host [15]. Codon usage is one of the most important features of sequence signatures. Since transmissible plasmids may exist in a wide range of hosts, their codon usage may exhibit mixed patterns in different hosts. Therefore, we surmise that the usage of synonymous codons of each amino acid on transmissible plasmids is more random than on non-transmissible plasmids. Based on information theory, we constructed a statistic named information entropy of amino acid (IEA) for 19 amino acids (i.e. 18 amino acids with more than 1 codon and 1 hypothetical stop amino acid corresponding to 3 stop codons) to assess the randomness of the synonymous codon usage on each plasmid genome. The IEA for a certain amino acid is defined as:

$$IEA{}_{i}=\sum_{j=1}^{n_{i}}p_{ij}\;log_{n_{i}}\frac{1}{p_{ij}}$$

where *i* represents a certain amino acid number (1≤*i*≤19); *j* represents a certain synonymous codon number of amino acid *i*; *p*_*ij*_ represents the frequency of use of the synonymous codon *j* for amino acid *i* in a certain plasmid genome; and *n*_*i*_ represents the number of synonymous codons of amino acid *i*.

Note that the IEA is a statistic between 0 and 1. In the field of information theory, information entropy is used to evaluate the uncertainty of a system. For a certain amino acid, the more random its usage of synonymous codons is, the higher the IEA. In particular, when the usage of synonymous codons follows a uniform distribution, namely, *p*_*ij*_==1/*n*_*i*_, the corresponding IEA achieves the maximum value of 1.

To calculate the IEA of each amino acid for each plasmid genome, we downloaded 4602 plasmids with transmissibility annotation from Shintani *et al*. [12] (referred to as the Shintani dataset henceforth). The 178 plasmid genomes labelled with a question mark (?) were removed from the dataset.

### PlasTrans design

In addition to synonymous codon usage, transmissible plasmids may also obtain other complex sequence signatures that are different from those of non-transmissible plasmids during the evolutionary process, such as di-codons usage, but many differences may be difficult to describe quantitatively. Recently, deep learning techniques have shown a strong ability to extract complex features from raw sequences [16] and therefore may be a powerful way to identify transmissible plasmid fragments.

**Fig. 1.**
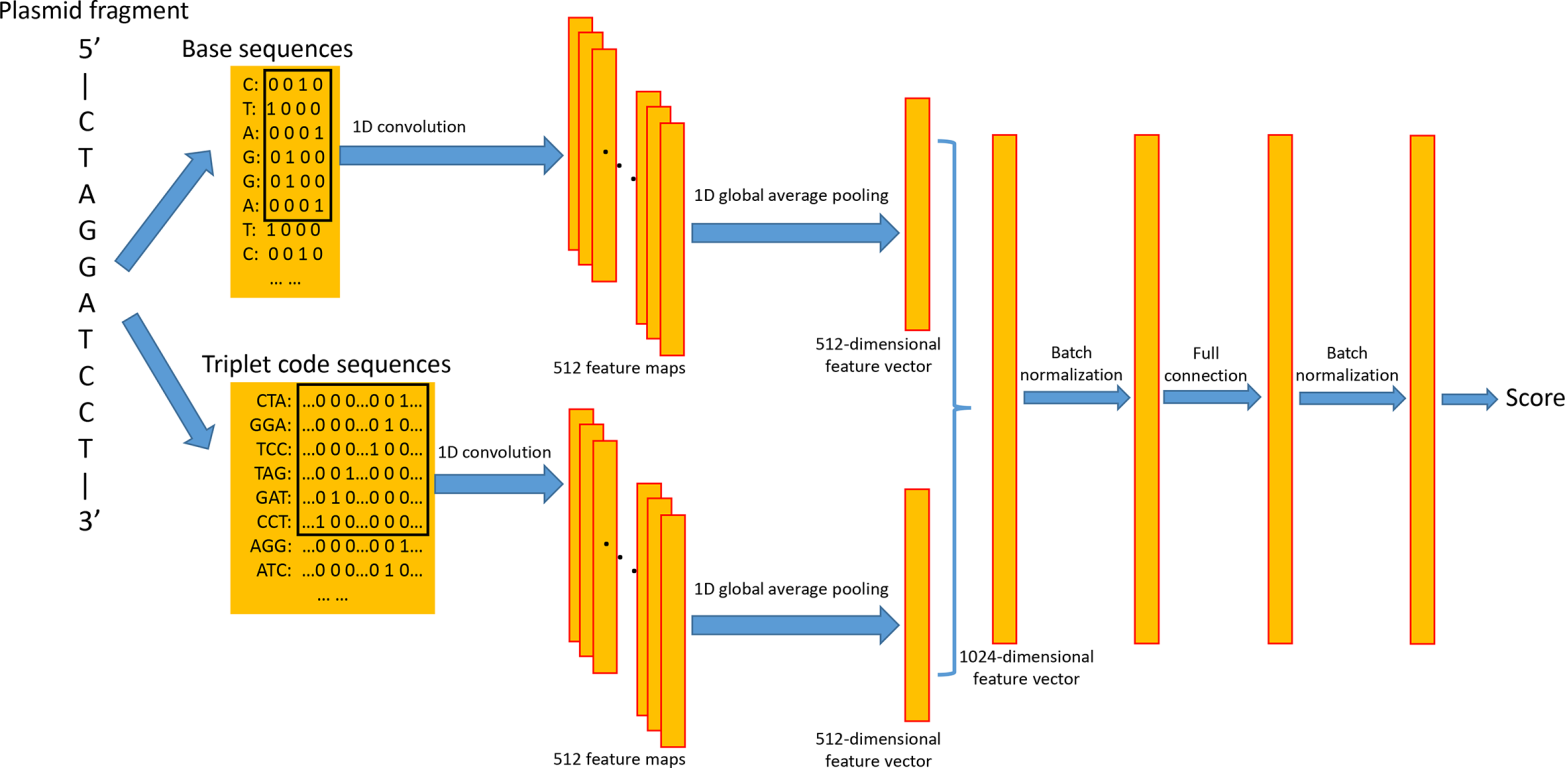
Structure of PlasTrans. PlasTrans takes the triplet code sequences and base sequences of a plasmid DNA fragment as input and provides a likelihood score that reflects whether the fragment is derived from a transmissible plasmid.

The architecture of PlasTrans is shown in Fig. 1. To better characterize the codon sequence, an input DNA fragment is expanded into six triplet code sequences. For example, the DNA sequence 5′-GCATTACGGCA-3′ is expanded to the following six triplet code sequences: (1): 5′-GCA, TTA, CGG-3′; (2): 5′-CAT, TAC, GGC-3′; (3): 5′-ATT, ACG, GCA-3′; (4): 5′-TGC, CGT, AAT-3′; (5): 5′-GCC, GTA, ATG-3′; (6): 5′-CCG, TAA, TGC-3′. These six triplet code sequences then connect to one triplet code sequence. In general, for a DNA fragment, only one of the six triplet code sequences belongs to the coding sequence (CDS). However, considering that gene prediction in plasmid fragments is relatively challenging and may miss some true positive predictions [14], we make full use of all six triplet code sequences to avoid missing important information. We used a one-hot encoding form to encode each triplet. Each one-hot vector contains 64 bits, in which 63 bits are 0 and a certain bit is 1. For example, triplet code AAA can be represented by the vector [1,0,0,…,…,0,0,0], triplet code AAC can be represented by the vector [0,1,0,…,…,0,0,0], and so on.

In addition to sequence signatures from the codon region, the non-coding region may also contain some specific motifs that may be helpful for identification, such as the *oriT*. To better characterize the sequence signatures from the non-coding sequences, the input DNA fragment is also represented by two connected base sequences (positive strand and complementary strand), in which the bases A, C, G and T are represented by [0,0,0,1], [0,0,1,0], [0,1,0,0] and [1,0,0,0], respectively.

For the triplet code sequences, PlasTrans employs a one-dimensional convolution operation to extract the sequence signatures. This convolutional layer contains 512 kernels, using ‘ReLU’ as the activation function. The kernel length is set to 6. After the convolutional layer, a total of 512 feature maps are generated, and a one-dimensional global average pooling layer further generates a 512-dimensional vector that describes the abstractive feature of the triplet code sequences. Similarly, a one-dimensional convolution operation, which contains 512 kernels with a length of 6 and uses ‘ReLU’ as the activation function, is employed to extract the sequence signatures from the base sequences. After the convolutional layer, a one-dimensional global average pooling layer generates a 512-dimensional feature vector from the feature maps for the base sequences. Two 512-dimensional feature vectors are then concatenated to form 1024-dimensional feature vectors. After three layers of batch normalization, full connection and batch normalization, the output layer calculates a score between 0–1, and input DNA fragments with a higher score have a higher possibility of belonging to the transmissible plasmid. In the training phase, PlasTrans used ‘Adam’ as the optimizer.

Given that real plasmidome data contain a large number of novel plasmids and cannot serve as a benchmark dataset, we trained and tested PlasTrans using artificial contigs extracted from well-annotated plasmid genomes in the Shintani dataset. Dividing the training set and test set according to the data release time is a commonly used method to evaluate whether an algorithm can work well on novel species [5, 7, 14, 17], which is also important for plasmidome studies. In PlasTrans, plasmids released before 2013 were used to construct the training set, while the rest were used to construct the test set (see Table S1 for the accession list). We used MetaSim [18] to randomly extract artificial contigs from the plasmid genomes. The sequence length of the training set was between 100 and 400 bp. In the test set, we generated sequences of four groups with different lengths: group A, 100–400 bp; group B, 401–800 bp, group C, 801–1200 bp; group D, 5000–10 000 bp. PlasTrans was trained using fragments of 100–400 bp, and for sequences longer than 400 bp in the prediction process, PlasTrans uses a scan window of 400 bp to move across the sequence without overlap. PlasTrans predicts each subsequence under the scan window independently and calculates the average score for the whole sequence. In the training set, we generated 300 000 sequences for both the transmissible plasmid and non-transmissible plasmid. In the test set, the number of sequences for transmissible and non-transmissible plasmids was 30 000 in groups A, B, and C and 10 000 in group D.

## Results

### The choice of synonymous codon for transmissible plasmids is more random

To quantitatively analyse whether transmissible plasmids contain different sequence signatures from the non-transmissible plasmids, we first calculated the IEA of each amino acid for each plasmid genome in the Shintani dataset. Interestingly, we found that the average IEA of all 19 amino acids in transmissible plasmids was higher than that in non-transmissible plasmids and the two-sided Wilcoxon rank sum test showed that the differences were significant (*P* value <0.05, see Table 1). In particular, the IEAs of transmissible plasmids are generally higher than 0.9, while those of non-transmissible plasmids are generally lower than 0.9. We further used each of the 19 IEAs to classify the transmissible and non-transmissible plasmid genomes, and the results showed that all IEAs could generally achieve an area under curve (AUC) >60 % (see Table 1), indicating that the choice of synonymous codon for transmissible plasmids is more random than that for non-transmissible plasmids. We consider that this phenomenon is caused by genome amelioration. It has been shown that bacteria of different species contain different sequence signatures [19], and during co-evolution, plasmids adjust their sequence signatures based on their hosts [15]. The similarity of sequence signatures between foreign DNA, such as plasmids and phages, and bacterial chromosomal DNA has also been widely used to predict the hosts of foreign DNA [15, 20, 21]. Since transmissible plasmids may exist in a wide range of hosts through conjugation, their genomes contain different sequence signatures, such as codon usage signatures, from different hosts. Therefore, the sequence signatures of transmissible plasmids are more complex and orderless. In terms of codon usage, such complexity and disorder may be reflected in the randomness of the choice of synonymous codon, leading to the higher IEA in transmissible plasmids.

**Table 1.** The average IEA of 19 amino acids on transmissible and non-transmissible plasmid genomes and the AUC for the use of a certain IEA to classify the transmissible and non-transmissible plasmid genomes. The *P* values of the two-sided Wilcoxon rank sum test showed that the IEA differences were significant

Amino acid	Average IEA (%)		AUC (%)	*P* value
	Transmissible plasmid	Non-transmissible plasmid		
K	88.58	84.56	57.05	1.24e−13
N	94.57	85.55	64.45	3.85e−52
T	93.73	88.16	62.21	9.35e−38
R	90.33	84.40	64.93	1.42e−55
S	94.17	90.07	64.56	6.70e−53
I	87.92	86.39	53.40	3.52e−4
Q	82.81	81.08	55.65	2.93e−9
H	96.07	88.13	64.72	4.67e−54
P	91.99	87.79	62.32	2.08e−38
L	84.10	83.15	53.15	9.18e−4
E	95.10	85.92	63.23	5.03e−44
D	94.03	84.27	65.49	1.19e−59
A	95.41	89.27	66.09	3.48e−64
G	94.41	92.48	58.24	4.40e−18
V	93.18	88.52	63.28	2.48e−44
Y	95.18	85.01	64.05	2.15e−49
C	91.83	88.30	55.94	4.19e−10
F	88.00	78.89	62.38	9.87e−39
Stop amino acid	85.35	81.95	55.82	9.30e−10

This phenomenon also provides biological support for the idea that the sequence signatures between transmissible and non-transmissible plasmids are different, indicating that fragments from these two kinds of plasmids can be distinguished by using sequence signature information, rather than information regarding the existence of specific genes.

### PlasTrans performance

**Fig. 2.**
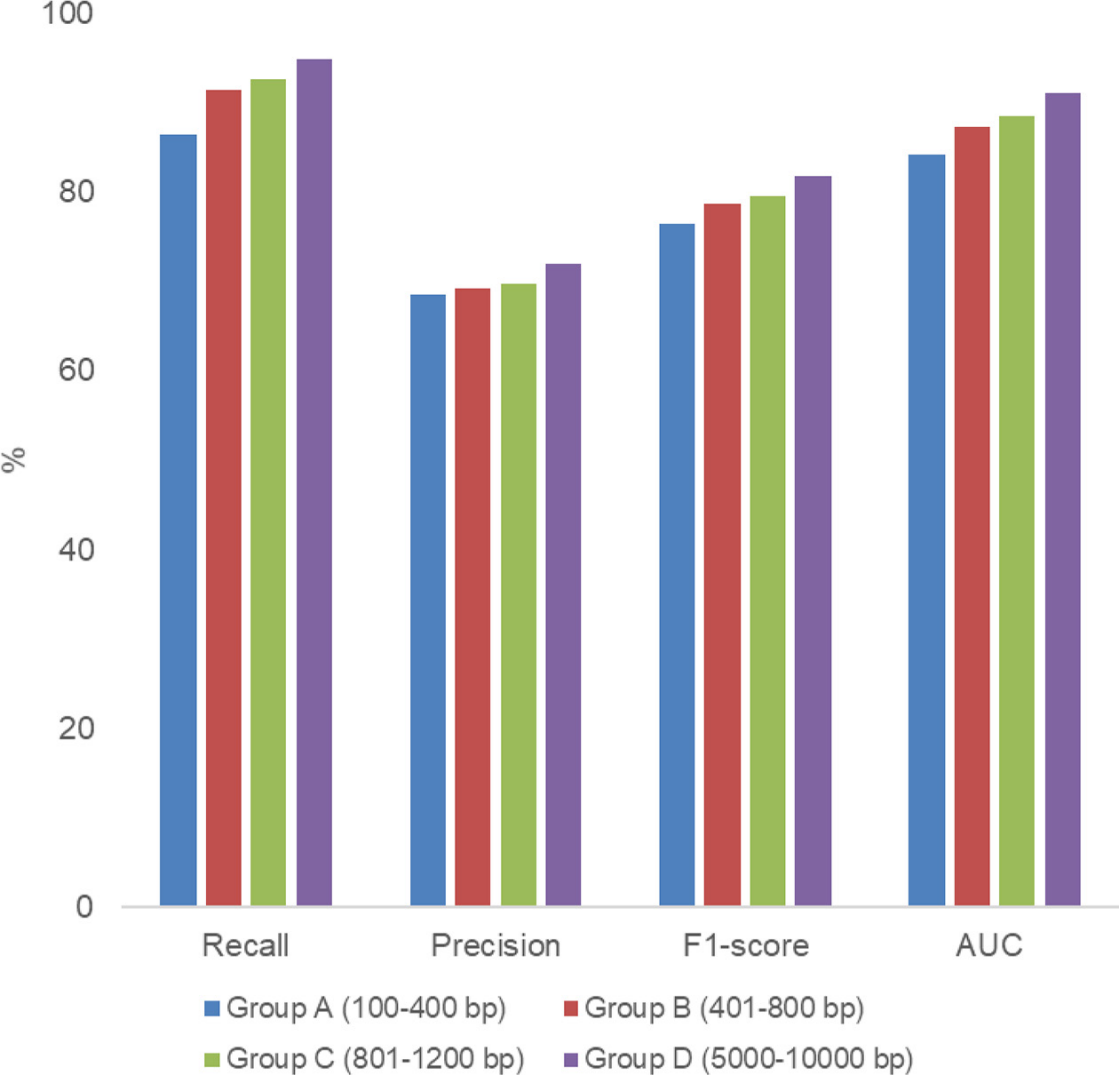
PlasTrans performance. PlasTrans was evaluated based on the benchmark test set of artificial plasmid contigs in different groups separately using the criteria of recall, precision, F1 score and AUC.

We first evaluated PlasTrans performance on the benchmark test set of artificial plasmid contigs. PlasTrans takes a plasmid fragment as input and provides a likelihood score between 0–1. By default, fragments with a score higher than 0.5 are considered transmissible plasmid-derived fragments (i.e. positive prediction). The evaluation criteria were *recall*=*TP*/(*TP+FN*), *precision*=*TP*/(*TP+FP*), *F1 score*=2×*recall*×*precision*/(*recall+precision*) and the AUC. We evaluated PlasTrans in different groups separately, and the results are shown in Fig. 2. In general, PlasTrans shows slightly better performance when the sequence is longer. For sequences with different lengths, PlasTrans was able to achieve satisfactory performance with an F1 score of approximately 77–82% and an AUC of approximately 84–91 %. Notably, sequences with lengths of hundreds of bases contain only a few genes or even incomplete genes. Therefore, this performance indicates that PlasTrans can extract the complex sequence signatures from the plasmid fragment effectively and judge the sequence bypass using the information for specific marker genes such as the relaxase gene. Considering the difficulty of the sequence assembly of plasmid metagenomic short reads, PlasTrans is suitable for analysis of plasmidome data. Further, some studies have shown that assembled contigs may lose some genes because of misassembly of plasmid raw reads; therefore, analysing plasmidome data using short reads directly may be more useful in some cases [14]. Given that PlasTrans was able to generate reliable results for sequences of different length, it may be a powerful tool to analyse different kinds of plasmidome data, such as raw reads and assembled contigs.

Moreover, we found that PlasTrans was able to achieve a satisfactory recall of approximately 86–95 %, but its precision was relatively low (approximately 70 %). This means that the predictions of PlasTrans contain some false positives. In our opinion, some of the false-positive predictions of PlasTrans may actually be true-positive predictions. In the Shintani dataset, the transferability of each plasmid genome is primarily annotated based on the existence of marker genes, such as the relaxase gene and some genes related to the conjugation process. Because of the diversity of plasmids, plasmid genomes contain a large number of novel genes with low similarity to the current gene database, and their function is unknown. In our previous work, we found that approximately 40 % of the genes in plasmid genomes from the RefSeq database have uncertain functions, which were labelled as either ‘putative’ or ‘hypothetical’, or lacked the exact product annotation [14]. Therefore, the Shintani dataset might miss some true-positive annotations because some functional marker genes for transmissible plasmids could not be found by searching the current database. Therefore, we considered that the precision of PlasTrans may be slightly underestimated.

It is worth noting that some transmissible plasmids exhibit a very narrow host range and the sequence signatures of these plasmids may be more similar to those of non-transmissible plasmids. To test whether PlasTrans will misjudge these plasmids as non-transmissible plasmids, we further collected the transmissible plasmids belonging to the incompatibility groups of IncF, IncH and IncI, whose host ranges were generally narrow [15], from the test set. From the artificial contigs extracted from these plasmids, PlasTrans was able to identify 95.62, 98.38, 98.72 and 99.3 % as transmissible plasmids in groups A, B, C and D, respectively. Such a high recognition rate indicates that PlasTrans identify transmissible plasmids with a narrow host range effectively.

**Fig. 3.**
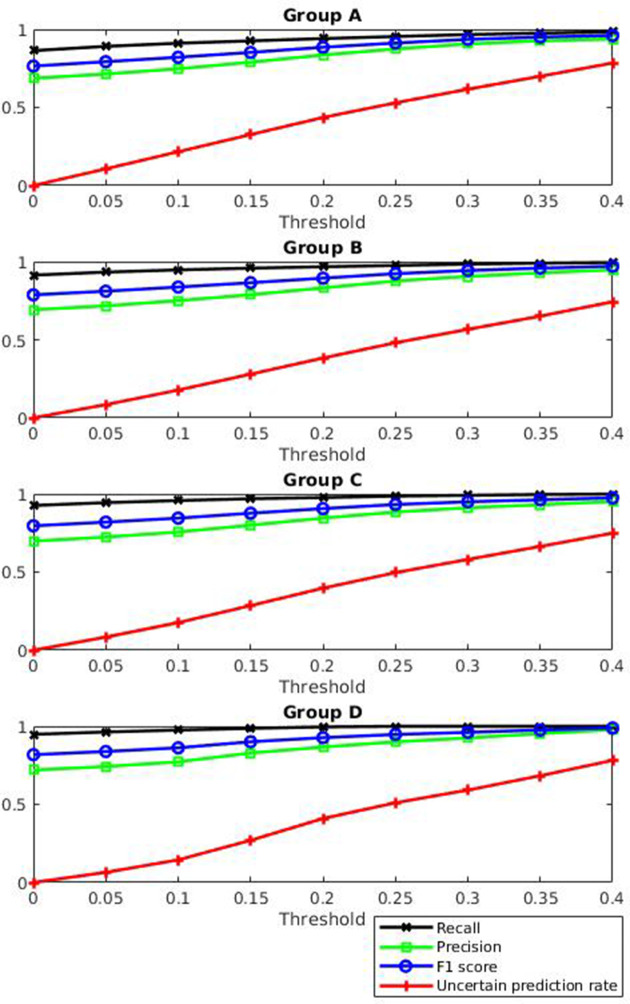
PlasTrans performance with different thresholds. Under a given threshold, *t*, a sequence with a score falling into the interval of |score−0.5|<*t* will be labelled as uncertain. Uncertain prediction rate, recall, precision and F1 score values of PlasTrans with different thresholds are evaluated.

To allow PlasTrans to generate more reliable results, the tool was designed with a parameter to filter out some uncertain predictions. The user can specify a threshold *t* (0<*t*<0.5); in this way, a sequence with a score falling into the interval of |score−0.5|<*t* will be labelled as uncertain. We evaluated the uncertain prediction rate, recall, precision and F1 score of PlasTrans with different thresholds, and the recall, precision and F1 score were calculated using only certain predictions. The results are shown in Fig. 3. The higher the threshold is, the higher the uncertain prediction rate, and the higher the recall, precision and F1 score of certain predictions. In particular, with a threshold of 0.2, the recall, precision and F1 score are higher than 80%, and approximately 60 % of the predictions are certain predictions.

We also evaluated PlasTrans qualitatively using real plasmidome data. We downloaded the assembled contigs of the bovine rumen plasmidome [1] from MG-RAST [22] (accession number: mgm4460391.3). This dataset contains 5771 contigs with a total of 2 710 501 bp. We first used PPR-Meta [7] to filter out contigs that may be chromosomal and phage DNA contamination. A total of 18.04 % of the contigs were predicted as chromosomal DNA, and 49.18 % of the contigs were predicted to be phage DNA. We then used PlasTrans to predict the transferability of the remaining contigs. We found that 61.52 % of the contigs were predicted to be transmissible, while the rest were predicted to be non-transmissible. To evaluate the reliability of the predictions, we collected the relaxases from the RefSeq plasmid protein database by searching for the keyword ‘relaxase’. We used blastx to search all contigs against the relaxases, and eight contigs that may contain the relaxase (e-value<=1e−3, hit length>=250) were collected. Among these eight relaxase-like contigs, six were predicted by PlasTrans to be transmissible, indicating that the prediction of PlasTrans was reliable. Among the two relaxase-like contigs that were not identified as transmissible plasmids, we found that PlasTrans gave them scores of 0.4972 and 0.4664, values quite close to 0.5. This indicated that these misjudgements could easily be filtered by setting a small threshold of *t*. Notably, in the training process of PlasTrans, the algorithm did not employ information concerning the existence of certain genes such as the relaxase gene; therefore, we consider that reliable prediction for relaxase-like contigs is not caused by the preference of the algorithm and can reflect the overall performance of PlasTrans.

## Discussion

To the best of our knowledge, PlasTrans is the first tool that can perform transferability annotation for short plasmid fragments in the plasmidome effectively using sequence signatures. The theoretical basis of PlasTrans is that transmissible plasmids contain different sequence signatures from non-transmissible plasmids, which we have proven in terms of synonymous codon usage using the IEA. PlasTrans bypass using the information of specific marker genes for judgement because such a strategy does not work for short fragments in the plasmidome. On the other hand, third-generation sequencing technology is increasingly being used in metagenomic studies, and it can generate very long contigs of the genome. In such cases, combining the usage of sequence signatures and the existence of specific marker genes may be a good choice for improving PlasTrans performance in the future because longer sequences have a higher probability of covering marker genes.

Transferable plasmids might have a broad or narrow host range, and the sequence signature between these two kinds of plasmids may also be different. Since we found that the choice of synonymous codon for transmissible plasmids is more random than that for non-transmissible plasmids, we also further surmised that the codon usage for transmissible plasmids with a broad host range is more random than that for a narrow host range and such differences may help us to predict the plasmid host range in the future study. Using the plasmid genome in the test set, we calculated the IEA of plasmids belonging to the incompatibility groups of IncF, IncH and IncI, whose host ranges were generally narrow, and plasmids belonging to the incompatibility groups of IncN, IncP and IncW, whose host ranges were generally broad [15]. Unfortunately, we found that the IEA of many plasmids with a narrow host range was not lower or even higher than that for those with a broad host range; only eight amino acids (K, Q, H, L, E, D, A and the stop amino acid) from plasmids with a narrow range were lower than those from plasmids with a broad host range, and among these eight IEAs, only the IEAs of Q and E showed a significant difference (Wilcoxon rank sum test, *P*<0.05), indicating that the codon usages of broad-host-range plasmids are not more random than those of narrow-host-range plasmids, which is inconsistent with our supposition. In our opinion, this is because codon usage in isolated plasmids may be slightly different from that in the plasmids in the microbiome community. The complete plasmid genomes we used in this study were from the National Center for Biotechnology Information (NCBI) database, with most of them being obtained in culture-dependent experiments, in which the bacterial host is isolated. In culture-dependent environments, horizontal gene transfer may be less frequent because in these environments, the selected bacterial strain does not need to face competition or cooperation as it does in natural environments, and the environment of a selective medium does not change; therefore, broad-host-range plasmids may lose some randomness of codon usage. Therefore, it is also important to analyse plasmid sequence signatures that bypass the culture-dependent experimental procedure in future studies.

When using PlasTrans to analyse the plasmidome data, users should pay attention to the purity of the plasmidome data if the plasmid DNA is enriched by experimental methods. In many cases, plasmidome data may contain chromosomal and phage DNA contamination. In particular, a large number of phages survive as circular DNA elements. When collecting plasmid DNA from the environment sample, these phages will also be collected together with the plasmids [7]. Therefore, we recommend the use of related tools, such as PPR-Meta [7], to filter out chromosomal and phage DNA contamination before using PlasTrans.

Another interesting finding was that PlasTrans could also identify some other mobilizable elements as transmissible DNA despite these elements not being plasmids. We used PlasTrans to predict the genomic island sequences in the Islander database [23], and we found that 68.68 % of the sequences were identified as transmissible DNA. We also used PlasTrans to predict two genomic island sequences of the SGI-1 family (NCBI accession numbers: KU847976.2 and AY463797.8) that exhibit a reasonably broad host range, and we found that PlasTrans could predict both of the sequences as being transmissible DNA. These results show that all mobilizable elements may contain some universal sequence signatures and PlasTrans can extract these features effectively. Such phenomena may also provide insights into the identification strategy of all mobilizable elements in the microbial community in future studies.

To date, many plasmidome-specific bioinformatic tools have focused on plasmid sequence identification or plasmid reconstruction, while tools for downstream analysis for detailed plasmid characterization are lacking, which prevents us from increasing our understanding of plasmid function in microbial communities. It is therefore expected that PlasTrans will be a powerful tool for plasmid characterization and classification in plasmidome studies.

## Supplementary Data

Supplementary material 1
